# Comprehensive hepatotoxicity prediction: ensemble model integrating machine learning and deep learning

**DOI:** 10.3389/fphar.2024.1441587

**Published:** 2024-08-21

**Authors:** Muhammad Zafar Irshad Khan, Jia-Nan Ren, Cheng Cao, Hong-Yu-Xiang Ye, Hao Wang, Ya-Min Guo, Jin-Rong Yang, Jian-Zhong Chen

**Affiliations:** ^1^ College of Pharmaceutical Sciences, Zhejiang University, Hangzhou, China; ^2^ Polytechnic Institute, Zhejiang University, Hangzhou, China

**Keywords:** hepatotoxicity, ensemble model, molecular fingerprints, machine learning, deep learning

## Abstract

**Background:**

Chemicals may lead to acute liver injuries, posing a serious threat to human health. Achieving the precise safety profile of a compound is challenging due to the complex and expensive testing procedures. In silico approaches will aid in identifying the potential risk of drug candidates in the initial stage of drug development and thus mitigating the developmental cost.

**Methods:**

In current studies, QSAR models were developed for hepatotoxicity predictions using the ensemble strategy to integrate machine learning (ML) and deep learning (DL) algorithms using various molecular features. A large dataset of 2588 chemicals and drugs was randomly divided into training (80%) and test (20%) sets, followed by the training of individual base models using diverse machine learning or deep learning based on three different kinds of descriptors and fingerprints. Feature selection approaches were employed to proceed with model optimizations based on the model performance. Hybrid ensemble approaches were further utilized to determine the method with the best performance.

**Results:**

The voting ensemble classifier emerged as the optimal model, achieving an excellent prediction accuracy of 80.26%, AUC of 82.84%, and recall of over 93% followed by bagging and stacking ensemble classifiers method. The model was further verified by an external test set, internal 10-fold cross-validation, and rigorous benchmark training, exhibiting much better reliability than the published models.

**Conclusion:**

The proposed ensemble model offers a dependable assessment with a good performance for the prediction regarding the risk of chemicals and drugs to induce liver damage.

## Introduction

The liver is a vital organ to oversee the metabolism and detoxification of essential compounds in the body. During the metabolic process of a compound, this organ is at substantial risk of impairment. All the compounds are xenobiotics to enter the body in the form of drugs, industrial chemicals, or environmental pollutants (Mohi-Ud-Din et al., 2019). Liver injury, commonly known as hepatotoxicity, can be caused by herbal remedies, dietary supplements, and organic solvents as well. Medicinal drugs usually produce hepatotoxicity in overdoses, and some may even induce liver damage in the therapeutic range. Medicinal agents account for 5% of hospital cases and 50% of acute liver failures (Abid et al., 2020). Drug-induced liver injury constitutes one of the primary factors leading to the failure of drugs during clinical trials. The Food and Drug Administration (FDA) has published a list of drugs causing liver injury, which is divided into four categories of Most-DILI concern, Less-DILI concern, No-DILI concern, and ambiguous DILI concern (Chen et al., 2016). It was reported that approximately 18% of drug candidates were withdrawn from the market or clinical trials due to the development of liver toxicity as a serious side effect from 1953 to 2013 (Andrade et al., 2019; Walker et al., 2020). Moreover, chemical-induced hepatotoxicity contributes to 30%–50% cases of acute liver failures (Mohi-Ud-Din et al., 2019). The mechanisms underlying hepatotoxic injury include inhibition of mitochondrial function, oxidative stress, activation of apoptosis, and disturbance of intracellular calcium homeostasis. Besides, many compounds such as carbon tetrachloride and paracetamol may have metabolic activations to form hepatotoxic metabolites, which is a process primarily facilitated by liver cytochrome P450 enzymes (Jaladanki et al., 2020; Pingili et al., 2023).

Evaluating chemicals for hepatotoxicity requires extensive safety studies involving the administration of chemical candidates to a large group of animals, thus incurring both time and financial costs (DiMasi et al., 2015; Tetko and tropsha, 2020). In many cases, animal models prove inadequate to accurately detect idiosyncratic hepatotoxicity, which may later manifest in humans during clinical trials or post-marketing surveillance (Dirven et al., 2021; Fernandez-Checa et al., 2021). In addition, *in vitro* techniques using high-throughput screening approaches have also been employed to assess drug toxicity. However, *in vitro* assays could be insufficient to fully substitute animal models since an individual *in vitro* assay primarily targets a simple toxicity mechanism (Raies and Bajic, 2016). Similarly, conventional computational models are inadequate and frequently prone to errors in forecasting complicated toxicity endpoints. These models are less robust to differentiate compounds with similar structure/chemical properties but manifest distinct toxicities (De et al., 2022). As discussed above, hepatotoxicity is complex, emanating from diverse mechanisms and different hepatotoxins. There is a significant need for the establishment of novel computational approaches and corresponding predictive models for hepatotoxicity that should be employed during the early phases of drug development.

Computational methodologies have been evaluated for regulatory decision-making, such as the REACH/3Rs framework implemented by European Union (EU) while projects like Tox21 and ToxCast by US Government agencies have made substantial advancements (Silva and Kwok, 2020; Jeong et al., 2022; Macmillan et al., 2023). These approaches have predominantly directed their efforts toward the assessment of high-throughput methodologies with a focus on methods that are based on *in vitro* assays, *in silico* procedures, and toxicogenomics for improving risk assessment. These methods leverage a variety of statistical approaches to create prediction models, but it is generally required of large and diverse datasets with well-documented toxicity outcomes. One approach is the use of artificial intelligence (AI), which has undergone swift evolution and paved the way for novel research and innovation, marked notably by the emergence of machine learning and deep learning (Xu et al., 2015). AI methods gained importance due to their ability to examine large datasets, make predictions, and offer insights into currently impossible events (Sharifani and Amini, 2023). Machine Learning (ML) and deep learning (DL) are the AI domains that have emerged rapidly, gaining a notable surge in recent years. ML automates the development of analytical models with data analysis methods, enhancing their performance from experience without the need for explicit programming (Mahrishi et al., 2020; Sharifani and Amini, 2023). DL constitutes a subset of ML, focusing on the employment of multilayer neural networks to extract the data in the categorized form for solving complex problems. DL models are inspired by the structure of the human brain to learn from the data in an automated way through its self-directed learning process. It is capable of training efficiently even with unstructured and complex data (Unterthiner et al., 2014; Hughes et al., 2015; Ma et al., 2015). The potential of ML and DL to revolutionize diverse industries and reshape the global landscape has become progressively evident with the ongoing expansion of generated data and the increasing computer power. The progress in these approaches will establish novel pathways for research and innovation, leading to significant breakthroughs in multiple fields like healthcare, transportation, and finance, etc (Alipanahi et al., 2015; Lee and Yoon, 2021). These approaches have capacities to facilitate a deeper understanding of complex systems, to enable informed decision-making, and to foster the development of more efficient as well as effective solutions to real-world problems (Park and Kellis, 2015; Li et al., 2020; Janiesch et al., 2021).

Quantitative structure-activity relationship (QSAR) is a computational modeling approach to predict the biological activity of chemical compounds by analyzing their molecular structure. QSAR models have the potential to identify compounds with desirable therapeutic activities or compounds with possible risks to human health in terms of absorption, distribution, metabolism, excretion, and toxicity (ADME/T) (Li et al., 2020). QSAR models developed by ML or DL algorithms can provide enhanced precision and robustness in predicting the bioactivities of chemical compounds. Couples of hepatotoxicity-predictive QSAR models have been developed so far, including a few studies of conventional ensemble models (Liew et al., 2011; Ai et al., 2018; Li et al., 2020). One thing common among these published models is based on drug-induced liver injury. To the best of our knowledge, none of these models included industrial compounds, chemicals, or organic solvents used in laboratories, which may also induce liver damage. In current studies, we enclose these organic compounds and solvents with known hepatotoxicity data to get a much larger dataset of 2588 organic compounds from different sources for generating a prediction model of hepatotoxicity in comparison to the previously reported works. Various base models were first created using each of five algorithms on three different molecular descriptors/fingerprints, i.e., RDkit molecular descriptors, Mordred descriptors, and Morgan fingerprints, respectively. The machine learning algorithms encompass 4 ML algorithms including support vector machines classifier (SVC), random forest classifier (RF), *K*-nearest neighbors (KNN), and extra tree classifier (ET), along with the DL algorithm of recurrent neural network (RNN). In order to improve the prediction capability of the generated model, voting ensemble strategy was further applied for the model development using the combination of above ML and DL base models on different molecular features. Ensemble model **I** was trained on base classifiers using RDkit molecular descriptors, ensemble model **II** was built on base models of Mordred descriptors, ensemble model **III** was trained on base models using RDkit molecular and Mordred descriptors together, and ensemble model **IV** was trained on base classifiers using Morgan fingerprints. As a result, the ensemble model **IV** trained on Morgan’s fingerprints emerged as the most effective among the evaluated models. The model exhibited an accuracy of 80.26% and an AUC score of 82.84%, outclassing all other models on hepatotoxicity. The model has a sensitivity of 93.02%, F1 score of 86.07%, and a precision of 80.19%. The ensemble model **IV** was further trained using 10-fold cross-validation to mitigate the risk of overfitting and to get more reliable predictions of the models. The ensemble model **IV** was further trained by including a second DL model, i.e., a multilayer perceptron (MLP) model. Later, RNN was removed and it was trained with MLP as the only DL model. Both models performed better to achieve AUC of around 80% but failed to outperform the model trained on RNN along with ML models. In addition, another two ensemble techniques of bagging and stacking were also applied for ensemble model **IV**, illustrating a lower performance than the voting ensemble classifier**.** In comparison to rigorous benchmark training performed on the descriptors and models presented in previously published studies, our generated models prove valuable for the identification of potential chemicals and medicinal drugs and could contribute to the reduction of hepatotoxicity-related attrition.

## Materials and methods

### Data collection, preprocessing and extraction of key descriptors

A diverse dataset of 2588 chemicals and drugs was assembled comprising compounds with documented evidence of hepatotoxicity or those without any reported hepatotoxic effects. Some compounds were gathered from the literature (Ai et al., 2018; Thakkar et al., 2020), containing the data of liver tox (Bjo¨rnsson. and Hoofnagle, 2016), Greene (Greene et al., 2010), Suzuki (Suzuki et al., 2010), and Xu data set (Xu et al., 2008). Besides, the liver-related toxicity data was mined from two public sites of comparative toxicogenomics database (The Comparative Toxicogenomics Database | CTD (https://ctdbase.org) and the National Library of Medicine (NLM,s) database (https://haz-map.com/heptox1.htm), enclosing industrial chemicals data in related to liver toxicity. RDKit package was utilized to get the canonical SMILES notation of the compounds from their chemical formula (Shi and Borchardt, 2017). After obtaining the canonical SMILES for each drug, all data sets were consolidated and duplicate entries were removed from the list. Mixtures and those lacking SMILES were removed from the dataset too. In addition, compounds with ambiguous activity against hepatotoxicity were excluded for further consideration. We chose to exclude all inorganic compounds and other mixtures from our model, keeping only organic compounds. It ensures data consistency and aids in dimensionality reduction because overfitting is more likely to occur in high dimensional data (Emmanuel et al., 2021). Compounds with known hepatotoxicity were labeled as 1 (positive) while those having no reported hepatotoxicity were labeled as 0 (negative). The dataset was finalized to contain 970 hepatotoxicity-negative compounds and 1618 hepatotoxicity-positive compounds, providing a balanced representation for analysis.

Numerical representation is an important depiction of the chemical compounds that can be used for multiple purposes. These representations capture different aspects of the molecular physico-chemical properties and are widely used in both cheminformatics and computational chemistry (Raghunathan and Priyakumar, 2022). Representing chemical data in a clear and precise manner that is easily understood by both humans and machines, enables tasks such as drug design, toxicology prediction, and material science research (Wigh et al., 2022). Depending on the internal chemical structure and external variables like shape, size, volume, and spatial context, there are various methods for molecular representation. These molecular representations play a pivotal role in the development of different ML and DL models by numerically encoding input data to facilitate its utilization in ML algorithms (Jaeger et al., 2018; Na, 2023). For every compound, two kinds of molecular descriptors, RDKit molecular descriptors and Mordred descriptors, were generated in addition to the Morgan fingerprints (1024 bits). RDKit molecular descriptors encompass a total of 208 molecular attributes retrieved from the PandasTools modules of the RDKit (Ji et al., 2023). Similarly, over 1800 Mordred descriptors were obtained for our dataset containing 1D, 2D, and 3D descriptors (Moriwaki et al., 2018). After computing descriptors, we excluded descriptors with missing values or non-numerical values, the majority of which were 3D descriptors, leaving a total of 1387 Mordred descriptors for model establishment. During the process of generating descriptors in machine learning and deep learning, few compounds may not produce descriptors to remain empty, and the missing values of the descriptors can cause reduced sensitivity during the process of model training (Yang et al., 2022). Moreover, a total of 2048 Morgan fingerprints were obtained from the RDKit library, which was subsequently utilized in the process of model building (Morgan, 1965). Table 1 illustrates the utilization of base models in conjunction with various descriptors and fingerprints to construct diverse ensemble models. The molecular descriptor values were normalized within the range between 0 and 1 using the MinMaxScaler function from the scikit learn package. In order to guarantee a well-balanced distribution of data and to facilitate a comprehensive evaluation of the model’s performance, the entire dataset was randomly split into two subsets: the training set included 80% of the compounds, while the test set had 20% of the compounds. Random splitting of the dataset is necessary to uphold a balanced and unbiased representation of compounds in each category (Ma et al., 2020).

**TABLE 1:** Construction of ensemble models based on different descriptors and fingerprints integrating ML and DL models.

Ensemble models	Method	Descriptor/Fingerprints used	ML base models	DL base models
Ensemble Model **I**	Voting	RDKit Molecular descriptor (208 in number)	SVC, KNN, RF, ET	RNN
Ensemble Model **II**	Voting	Mordred descriptors (1387 descriptors)	SVC, KNN, RF, ET	RNN
Ensemble Model **III**	Voting	RDKit + Mordred descriptors (1595 in number)	SVC, KNN, RF, ET	RNN
Ensemble Model **IV**	Voting	Morgan fingerprints (2048)	SVC, KNN, RF, ET	RNN, MLP

## Framework for base models development

**FIGURE 1:**
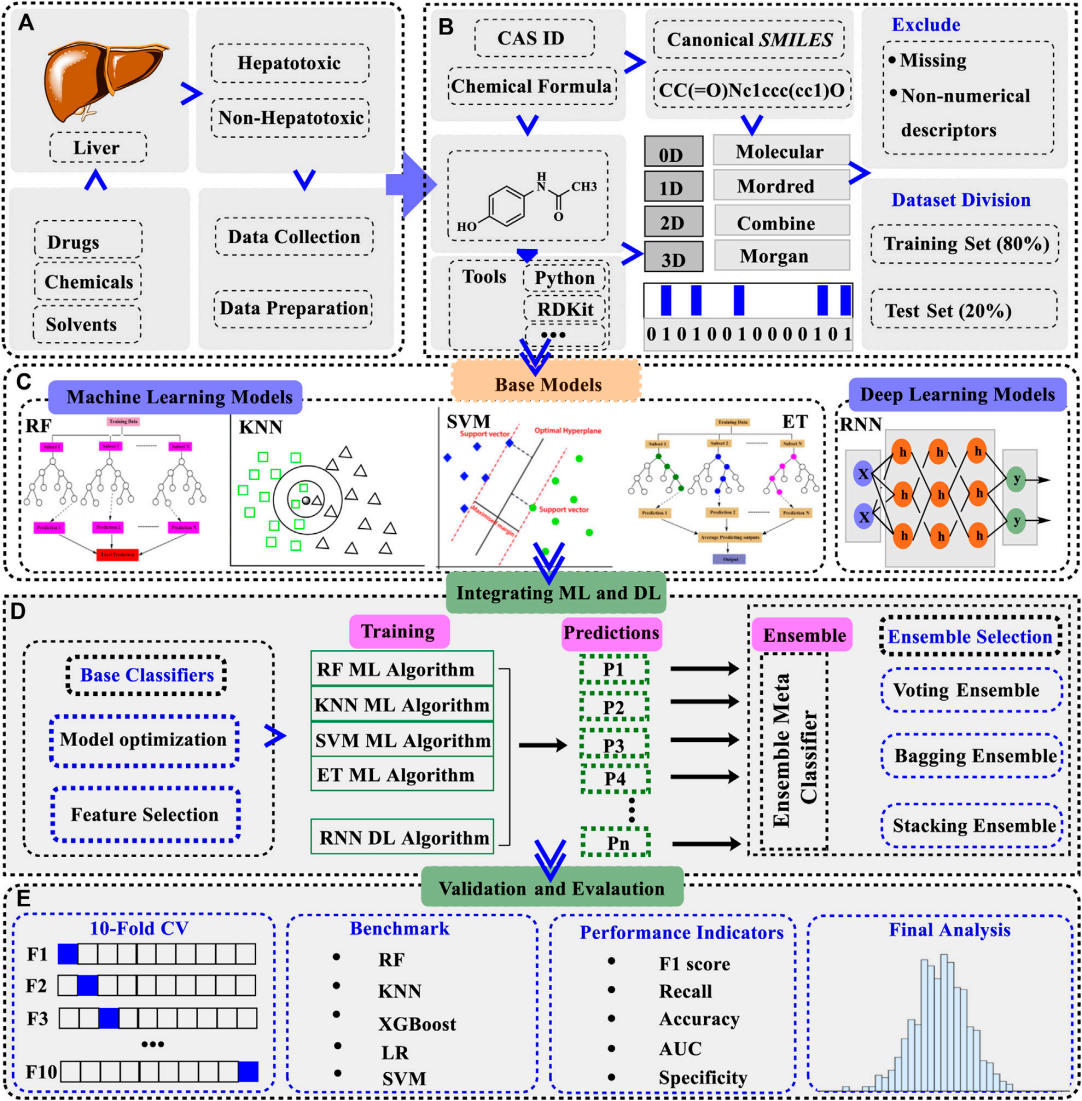
A schematic overview of the workflow of generating a hybrid ensemble model integrating machine learning (ML) and deep learning (DL) base classifiers **(A)**. Hepatotoxicity data was collected from the literature and online sources. The compounds were categorized into positive (hepatotoxic) and negative (non-hepatotoxic) based on their established attributes **(B)**. Different descriptors/fingerprints were downloaded for the dataset and compounds containing missing values were removed. The dataset was divided into training (80%) and test set (20%) **(C)**. ML and DL learning base models were trained on the dataset. Four ML models including RF, KNN, SVC, and ET were constructed initially along with the RNN as a DL model **(D)**. ML and DL mixed ensemble model was assembled from the individual base models after the feature selection process. Three different kinds of ensemble models were built to choose the one with the optimal performance **(E)**. Dynamic validation approaches were employed to verify the authentication of the models and the final evaluation was recorded in terms of accuracy, AUC, F1 score, recall, and specificity. Abbreviations: RF: random forest, KNN: K-nearest neighbors, SVM: support vector machines classifier, ET: extra tree classifier, RNN: recurrent neural network, LR: logistic regression, XGBoost: extreme gradient boosting, RF ML algorithm: random forest machine learning algorithm, KNN ML algorithm: K-nearest neighbors machine learning algorithm, SVM ML algorithm: support vector machine learning algorithm, ET ML algorithm: extra tree machine learning algorithm, 10-Fold CV: 10-fold cross-validation, AUC: area under the ROC curve.

We divided the model development process into five different stages as shown in Figure 1. Starting with the collection of data (Figure 1A) and the retrieval of descriptors (Figure 1B), as described above, the datasets were then trained on different ML and DL models to make individual base models. We built 4 ML models of SVC, RF, KNN, and ET while one DL model of RNN as the individual base model (Figure 1C). Next, the embedded feature selection (FS) process was followed by making a descriptors list ranked in ascending or descending order based on feature contributions. In this approach, a specific number of descriptors were sequentially removed each time from the set of descriptors and the performance of the model was recorded at each step. Here, model performance was evaluated by removing descriptors from the ranked list of descriptors as given in Equation 1.

$${model\;performance}_{i}=\frac{\;{AUC}_{w}-\;{AUC}_{i\;}}{\;{AUC}_{w}}$$ (1)

where “i” denotes the selected descriptor whereas “w” denotes the whole set of descriptors i.e., AUC_i_ represents the AUC value of the model developed without descriptor i, and AUC_w_ denotes the AUC value of the model developed using all the selected descriptors. A greater accuracy and higher area under the curve (AUC) suggest that the removed feature has less impact on the model performance.

### Fundamentals of ensemble models development

**FIGURE 2:**
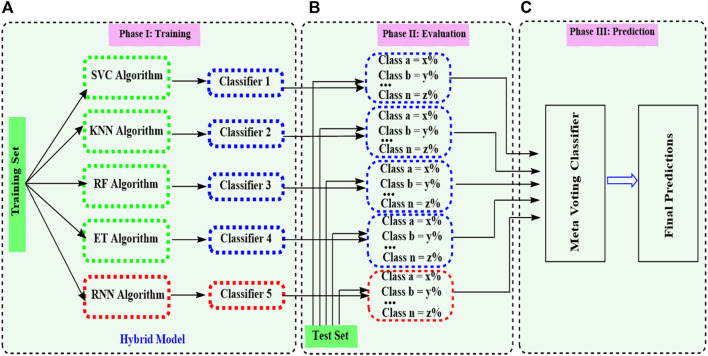
Ensemble model merging machine learning (ML) and deep learning (DL) models. The process consists of three stages **(A)**. In the initial phase, base models are trained on their training set. The hybrid model indicates a combination of ML and DL models. **(B)** In the second phase, the evaluation of each base model is done according to their performance on the dataset. Class a, Class b, …, and Class n represent different performances of each base model defined in section (A). **(C)** The voting ensemble model analyzes the base model calculations on which it has to make the final predictions.

After getting the optimized descriptors and fingerprints for the base models, ensemble modeling integrating ML and DL was established (Figure 1D). A hybrid ensemble was employed to integrate ML and DL models in a unified manner, ensuring equal contributions for all base models in the decision-making process. The architecture of the ensemble model integrating ML and DL is presented in Figure 2. A voting ensemble classifier was initially used to select the model with the optimal performance. Later bagging and stacking ensemble modeling were employed for the best model as the benchmark modeling. The optimal ensemble model was validated with a 10-fold cross-validation process (Figure 1E). In addition, the RNN base classifier of the optimal model was replaced with other DL models such as multilayer perceptron (MLP) to further validate the model. We also integrated MLP along with the RNN model and applied ensemble modeling using a voting classifier to confirm the integrated effect of multiple DL models with the multiple ML models. Finally, benchmark modeling was performed on our dataset with the descriptors and the base models published in the literature (Figure 1E).

## Uncovering the mechanics of RNN and MLP models

Recurrent neural networks (RNNs) are specialized networks for handling sequential and input-output data with varying lengths such as time series or sequences of data points (Nosouhian et al., 2021). RNN learnings are commanding sequential learners connected by three different layers including input layers, recurrent layers, and output layers. RNN modeling has garnered attention for model generations owing to its ability to address machine learning challenges. In our model, the RNN consisted of one implicit input layer, two recurrent layers of long short-term memory (LSTM) having 64 units, and one output layer, which is a dense layer with a sigmoid activation function. A dropout layer was added after the LSTM layer to provide regularization and prevent overfitting. The features of the second LSTM layer are similar to those of the first layer. A wrapper class (RNNWrapper) was created that implements the scikit-learn Base Estimator and Classifier Mixin interfaces. The trained RNN model was used to instantiate the wrapper class.

Multilayer perceptron (MLP) is a neural network having multiple layers of interconnected nodes, making it acquire complex patterns and relationships within data, making it a key component of deep learning architecture (Naskath et al., 2023). We instantiated an MLP classifier for our model with specific parameters. Our MLP models include one input layer, 2 hidden layers, and one output layer. The first hidden layer consists of 100 nodes followed by another layer of 50 nodes. The model consists of a maximum of 500 iterations for convergence and was adjusted to weights and biases iteratively by fitting to the training data leveraging the backpropagation algorithm.

### Models performance evaluation

The performance of the generated predictive models can be evaluated using different strategies. In general, if the ensemble model performs worse than base classifiers despite taking all aspects into account, this would violate the fundamental notion of ensemble modeling (Campagner et al., 2023). Therefore, the ensemble model is expected to have a reliability surpassing the base classifiers. Initially, it was compared to the performance of the ensemble model with the individual base models. We used both internal and external validation methods to assess the model performance. To generate more consistent and reproducible results, a 10-fold cross-validation was employed for internal validation. In this approach, the initial dataset is randomly partitioned into 10 equal subsamples during the 10-fold cross-validation. In each iteration of the process, 9 of these subsamples are employed as the training set while one subsample serves as the validation set to evaluate the performance of the model. The process is repeated ten times so that each subsample serves as a validation set once and hence, gives us a robust assessment of the model across different data subsets. The purpose of repeating this procedure is to reduce the influence of chance on the outcomes, ensuring a more reliable evaluation of the model. Internal validation assists in refining model parameters and mitigating the risk of overfitting. Moreover, external validation was used to assess the performance of the model on the external test set. External validation is used to evaluate the model performance on a dataset that is not exposed during the training of the model, providing a reliable measure of its generalization capabilities. This validation offers a more realistic assessment of the model performance in real-world scenarios because it is tested on an independent dataset that was not used during the model development. Furthermore, benchmark models were employed to compare the outcomes of the published studies with the result of our proposed ensemble model. The findings of published studies were compared with individual base models as well as with ensemble learning. We extracted the same descriptors/fingerprints used in the published articles for our dataset and subsequently trained our models for the same ML models used in their studies. The methodology employed was consistent with that utilized in our ensemble model.

Various metrics were used to evaluate the predictive performance of models (Equations 2–5). Five different key indicators were used to assess the performance of our model containing accuracy (Q), representing the overall prediction accuracy of both positive and negative samples of the model, sensitivity (SE), indicating the accuracy of predicting positive samples, reflecting the accuracy of predicting negative samples, and the area under the receiver-operating characteristics curve (ROC). Similarly, the F1 score (F1) was computed, which is thought to be a better performance indicator than the regular accuracy measure and precision (PRE), which correctly measures how much was correctly classified as positive out of all positive.

$$Accuracy=\frac{\;\text{TP}+TN\;}{\;\text{TP}+\text{TN}+\text{FN}+\text{FP}\;}\times 100$$ (2)

$$Sensitivity=\frac{\;TP\;}{\;\text{TP}+\text{FN}\;}\times 100$$ (3)

$$Precision=\frac{\;TP\;}{\;\text{TP}+\text{FP}\;}\times 100$$ (4)

$$F1=\frac{\;TP\;}{\;\text{Tp}+\frac{1}{2}\left(\text{FP}+\text{FN}\right)\;}\times 100$$ (5)

Here in the above formulas, true positive (TP) represents the number of positive (hepatotoxicants) samples correctly predicted by the model, true negative (TN) represents the number of negative samples (non-hepatotoxicants) correctly predicted by the model, false positive (FP) denotes the count of negative samples (non-hepatotoxicants) that are wrongly predicted as positive (hepatotoxicants), while false negative (FN) represents the number of positive samples (hepatotoxicants) that are wrongly predicted as negative (non-hepatotoxicants).

### Statistical analysis

The data presented in the results represents the mean value along with the standard deviation (SD), calculated from the repeated instances. One-way analysis of variance (ANOVA) was employed to identify statistically significant groups, with significance levels reported as *p* < 0.05.

## Results

A couple of studies have explored the development of hepatotoxicity prediction models, each leveraging a dataset of less than 1600 compounds on drug-induced liver injury. There are certain difficulties in the design of DL models to predict liver toxicity and one such complication is sample size. In this study, we collected an enlarged dataset of 2588 compounds including industrial compounds and solvents with known hepatotoxic or non-hepatotoxic effects along with the drugs. We used three different kinds of descriptors and fingerprints for building four different ML and two DL models on our dataset, as shown in Table 1. This was followed by different ensemble modeling to find the optimal model with the highest performance. The performance of the models was evaluated by an independent test data set and a 10-fold cross-validation. A detailed analysis of the predicted models was conducted to assess the prediction accuracy, F1 score, SE, and Area Under the Receiver Operating Characteristic Curve (ROC-AUC). Evaluation criteria are critical to finding a model that can identify and efficiently filter chemicals and drugs that pose a risk of liver injury as well as recognizing substances that are not associated with this risk.

### Development of ML and DL base models: commencement of liver toxicity prediction models

Liver toxicity prediction models were initially constructed individually with ML and DL algorithms using three different kinds of descriptors and fingerprints. After data preprocessing by removing mixtures and redundant compounds, the dataset was randomly divided into training and test sets followed by the model fitting on the individual descriptors. Initially, five different models were built including 4 ML and one DL models for each type of descriptor/fingerprint. All the models followed the same development strategy but they were built using different descriptors or fingerprints. The results of the base models constructed on different descriptors and fingerprints are presented in Supplementary Tables S1–S4. Among all, the model based on the Morgan fingerprints outperformed the other models in terms of performance. Most of the ML and DL models had a prediction accuracy below 0.70 on the test dataset with only a couple of the ML base models displaying accuracy higher than 0.70. For almost all the descriptors, we attained an accuracy of approximately 0.70 for the ML base models but none surpassing 0.75 on the test dataset. The performance of the DL RNN models was even inferior to that of ML models for all these descriptors. Among DL base models, the RNN model based on Mordered descriptors demonstrated the best performance with an accuracy of 0.72 (Supplementary Table S2).

As described above, all the base models trained on molecular features using individual machine learning/deep learning algorithms were unable to demonstrate satisfactory prediction capability. The ensemble strategy was further employed for these base classifier models but the performance of any ensemble model failed to meet the desired level of accuracy expected from an efficient model. We modified our approach and decided to use the feature importance step based on the significance of different available descriptors after finding that the performance of each of the individual base models was not adequate. Features were selected based on model contribution by placing them in ascending or descending order and then choosing the best optimal features among them to produce the model with the best performance. This approach is called sequential feature selection, where we gradually decrease or increase the selected features to find an interpretable model at the end (Li et al., 2020). The ultimate goal was to reduce the number of the informative descriptors to get a reliable relationship between the chosen descriptors and the target variable.

In the process of feature optimizations, descriptors were firstly arranged in descending order based on their contribution to the model. After that, we sequentially removed a certain number of descriptors each time to build a model on the remaining descriptors, checking the performance of the model. The aim was to eliminate the uninformative descriptors, minimizing the loss of prediction performance. As illustrated in Supplementary Tables S5–S7, the feature selection approach significantly enhanced the performance of almost all the ML and DL models. Enhanced performance was not limited to any specific models but all models built on different descriptors exhibited better performance compared to their previous one. After feature optimization, all the ML and DL base models demonstrated better performance with an accuracy of over 0.70 for all types of molecular descriptors/fingerprints. Meanwhile, it was indicated that models built on Morgan’s fingerprints outperformed all other models (Table 2), hence, emerging as the most effective model. The SVC base model built on Morgan’s fingerprints emerged as the best model among all the base models with an accuracy of 0.783 followed by the RNN base model. All the base models constructed on Morgan’s fingerprints displayed prediction accuracies of over 0.75 after following the feature importance approach. The results of the base models constructed on Morgan’s fingerprints are presented in Table 2.

**TABLE 2:** Performance metrics of individual base models and ensemble model **IV** built on Morgan fingerprints using the voting ensemble technique in comparison to other ensemble models. Results of the ensemble models presented here are expressed as mean value (n = 5).

Models	Accuracy	AUC	F1 score	Sensitivity	Specificity	Precision
**KNN**	0.7648	0.7521	0.8387	0.9195	0.3790	0.7710
**SVC**	0.7839	0.8103	0.8458	0.8908	0.5714	0.8051
**RF**	0.7686	0.8131	0.8371	0.8936	0.5200	0.7873
**ET**	0.7629	0.8180	0.8277	0.8563	0.5771	0.8010
**RNN**	0.7705	0.8088	0.8255	0.8160	0.6800	0.8352
**MLP**	0.7380	0.6870	0.7977	0.8390	0.5371	0.7828
**Ensemble model I**	0.7381	0.7893	0.8107	0.8521	0.5189	0.7657
**Ensemble model II**	0.7589	0.7910	0.8227	0.8613	0.5448	0.7867
**Ensemble model III**	0.7532	0.7921	0.8242	0.8713	0.5224	0.7808
**Ensemble model IV**	**0.8026**	**0.8284**	**0.8607**	**0.9302**	0.5424	0.8019
**Ensemble model IV(A)**	0.7823	0.8147	0.8323	0.8891	0.5579	0.7946
**Ensemble model IV(B)**	0.7791	0.8109	0.8407	0.8933	0.5381	0.7931

**Ensemble model I:** Ensemble model built on 4 ML, models (KNN, SVC, RF, ET) and one DL, model (RNN) using RDKit, Molecular descriptor.

**Ensemble model II:** Ensemble model built on 4 ML, models (KNN, SVC, RF, ET) and one DL, model (RNN) using Mordered descriptors.

**Ensemble model III:** Ensemble model built on 4 ML, models (KNN, SVC, RF, ET) and one DL, model (RNN) using RDKit, Molecular descriptor and Mordered descriptors together.

**Ensemble model IV:** Ensemble model built on 4 ML, models (KNN, SVC, RF, ET) and one DL, model (RNN) using Morgan fingerprints.

**Ensemble model IV(A):** Ensemble model built on 4 ML, models (KNN, SVC, RF, ET) and one DL, model (MLP) using Morgan fingerprints.

**Ensemble model IV(B):** Ensemble model built on 4 ML, models (KNN, SVC, RF, ET) and two DL, models (RNN, MLP) using Morgan fingerprints.

Note: Best scores are marked as bold.

The feature importance approach improves the performance of almost all the base models built on other descriptors. The improvement was not limited to the accuracy but was evident in all the predictive indicators of the models including SE, SP, F1 score, and AUC as well. The results of different base models utilizing the feature importance approach are given in Supplementary Tables S5–S7 (supplementary file). The performance of machine learning and deep learning models was assessed systematically using different hyperparameter conditions to acquire the best base models. The optimized conditions facilitated us to identify and select the most pertinent variables, ensuring the precise correlation of the chemical structures with their toxicological effects. Each model acquired independent optimization to get the best possible results for different training models. Base classifiers were set for each model with the optimal parameters, i.e., optimized number of neighbors K was 5 in the case of the KNN model. RF and ET models were trained on training data using 700, 500, 300, and 100 n_estimators to select and optimize the best estimators. Similarly, parameters for the RNN model were optimized after trying different settings, identifying that it performed finest using the learning rate of 0.001, 10 epochs, and batch size of 128. Selecting optimal features and hyperparameters enabled us to achieve the best results for our base models, paving the way for the development of ensemble learning.

### Ensemble strategies for converging ML and DL models

After concluding the features optimization for the individual base model development, we further proceeded with the ensemble modeling strategy, utilizing several ensemble techniques to merge the ML base models that were developed earlier with the DL base model. Ensemble algorithms were built on the individual ML and DL models to extract and integrate all the information produced from the individual models. We proposed a new ensemble learning technique trained on a blend of ML and DL models by integrating them together. The hybrid approach is anticipated to increase the performance and prediction ability of individual ML and DL models in determining the hepatotoxicity of drugs and chemicals.

**FIGURE 3:**
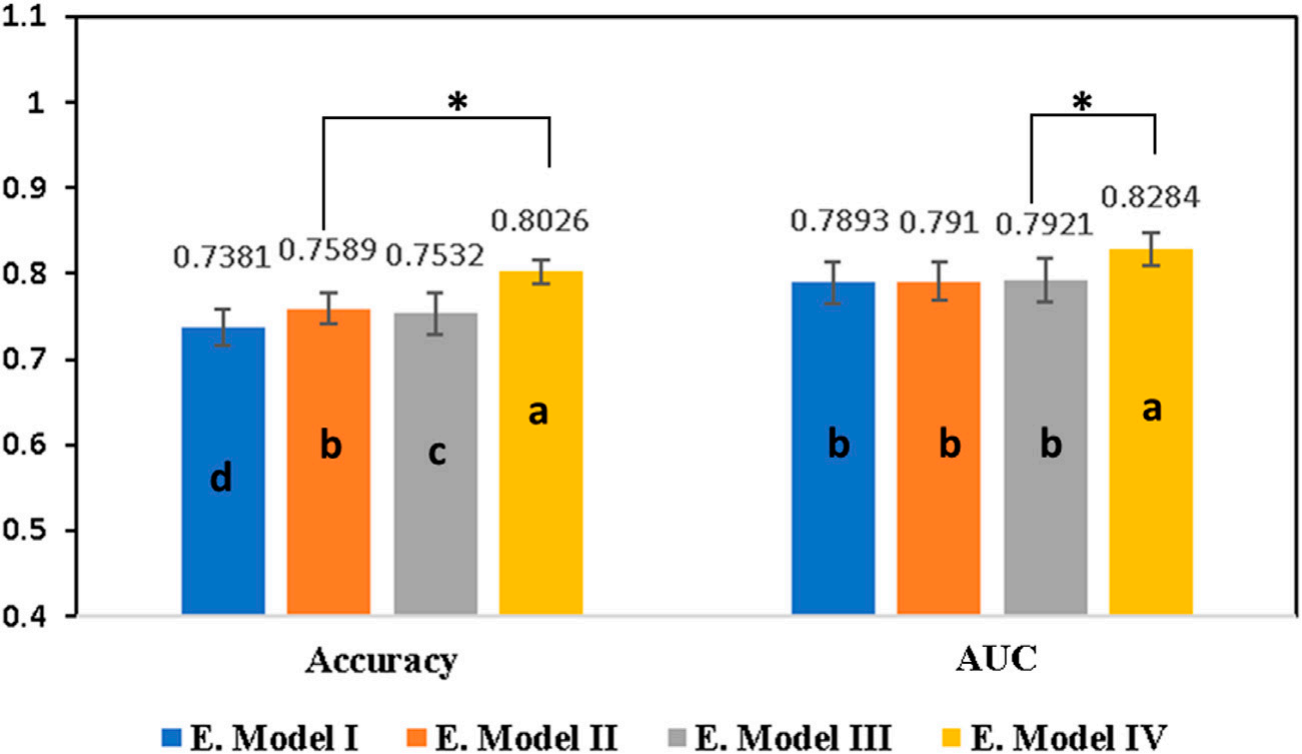
Comparison of all four ensemble models after employing the feature selection approach built on different descriptors and fingerprints using the voting ensemble technique. Ensemble model **I** was built on RDKit Molecular descriptor and ensemble model **II** was constructed using Mordered descriptors. Similarly, ensemble model **III** was built using RDKit Molecular descriptor and Mordered descriptors together while ensemble model **IV** was built on Morgan fingerprints. Results are expressed as mean ± SD, n = 5. Different letters **(A–D)** on the bars indicate significant differences among the different groups at *p* < 0.05 by one-way ANOVA with the Duncan test. The * represents the significance between the two top groups at *p* < 0.05. Typically, AUC is the area under the ROC curve, E. Model I represents the ensemble model I, E. Model II represents the ensemble model II, E. Model III represents the ensemble model III, and E. Model IV represents the ensemble model IV.

**FIGURE 4:**
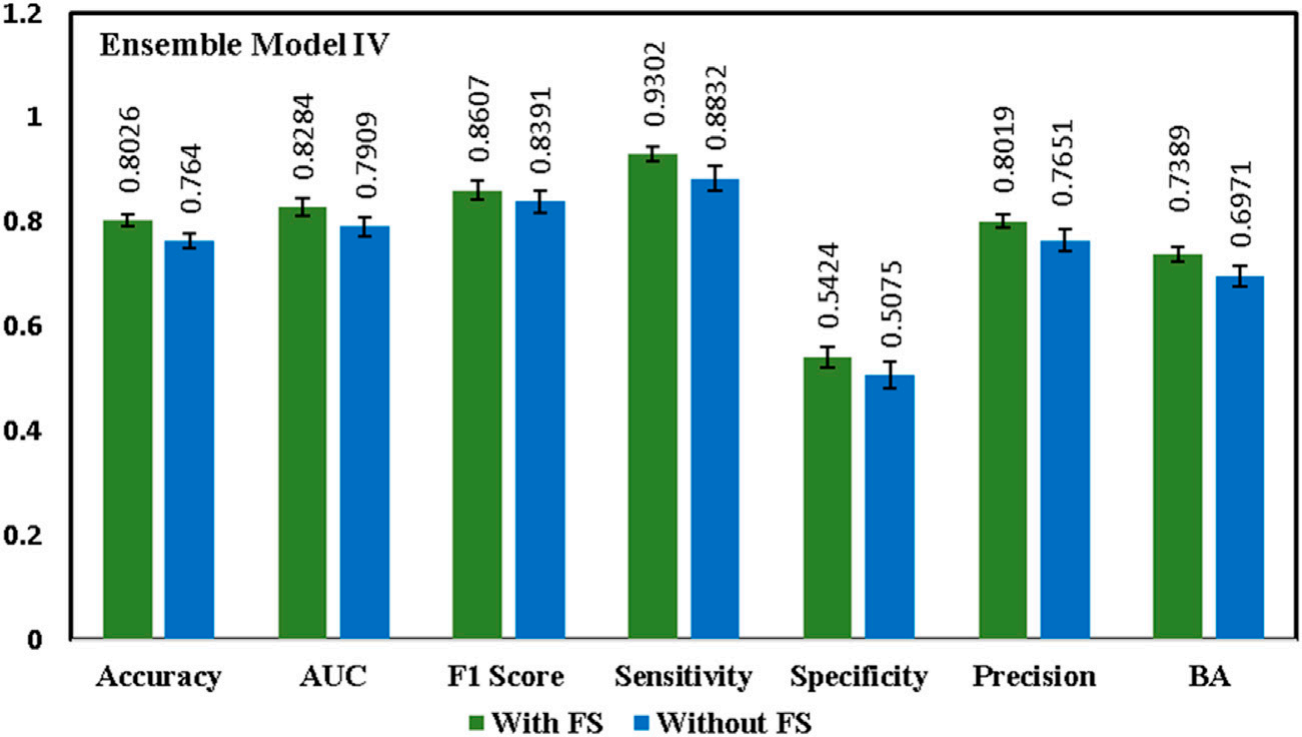
Comparison of the ensemble model **IV** parameters with FS (feature selection) and without FS (feature selection). Results are expressed as mean ± SD, n = 5. AUC is the area under the ROC curve and BA is the balanced accuracy.

Initially, a primary framework was constructed using the 4 ML base models incorporated together with the RNN base model. A voting ensemble classifier was used to build a primary framework for all four ensemble models (Table 1) based on the RDKit Molecular/Mordered descriptors or Morgan fingerprint. Voting classifier gathers predictions for each class label and directs class labels with the highest number of votes or largest probability for predictions (Kim et al., 2003). The ensemble model **IV** with Morgan fingerprints outperformed all other three ensemble models by achieving an AUC score of 82.84% and an accuracy of 80.26% (Table 2). Figure 3 illustrates performance comparisons of the ensemble model **IV** with and without the use of feature selection. In fact, the voting ensemble classifier makes the other three ensemble models **I**, **II**, and **III** to perform well by attaining an accuracy of 75% or above, as shown in Figure 4. In current studies, the hybrid ensemble method effectively mitigates classification uncertainty and improves the overall performance of the base classifiers. The different ensemble techniques vary from one another in how they train and integrate diverse baseline models (Krawczyk et al., 2017). Traditional ensemble models integrate conventional ML models, applied across different fields. Recent efforts have directed to extend the ensemble learning techniques to DL but still, there are limitations in the training and generalization of such models (Ammar and Kora, 2023). The better performance of the voting ensemble classifier can also be attributed to the robustness of the models, which effectively generalizes to the test set and prevents the overfitting of the model.

### Benchmarking, validation, and evaluation of optimal model performance

**FIGURE 5:**
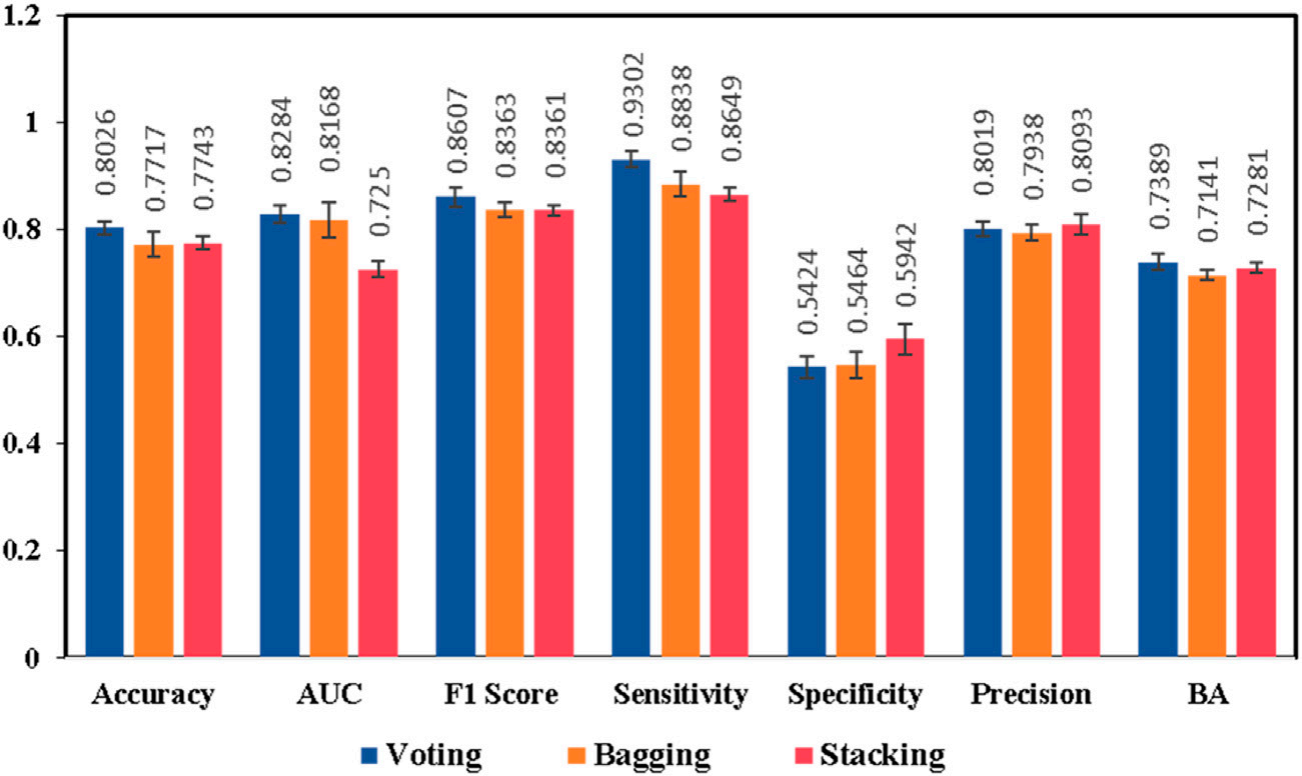
Performance comparisons of the ensemble model **IV** generated by different ensemble strategies, voting, bagging, and stacking ensembles, respectively. Results are expressed as mean ± SD, n = 5. AUC is the area under the ROC curve, BA is the balanced accuracy.

**FIGURE 6:**
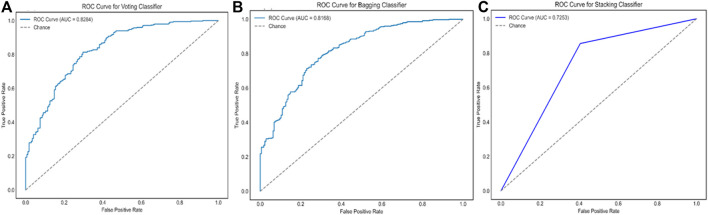
The ROC curves and the AUC scores of ensemble model **IV**, using three different strategies of the voting ensemble **(A)**, the bagging ensemble **(B)**, and the stacking ensemble **(C)**, respectively.

Keeping in view the intricate nature of combining different ML models with DL models, we proposed additional ensemble techniques for the model exhibiting the best performance, including bagging and stacking ensemble methods. The main objective was to design an ensemble method which is capable of capturing maximum drug and chemical-related information on liver-related toxicities to provide maximal predictive evidence with optimal efficacy. In current studies, both bagging and stacking models performed well to achieve better accuracies of 77.17% and 77.43% respectively, in comparison to the base models (Supplementary Tables S1–S3). The findings were consistent with the possibility that employing computational ensemble models with diverse molecular descriptors could lead to increased prediction performance (Mora et al., 2020). However, the results indicated that the bagging and stacking methods would not have a good performance as voting ensemble classifier for our model generations as illustrated in Figure 5 and Figure 6.

Next, we evaluated the performance of the best model (ensemble model **IV**) using a 10-fold cross-validation approach. The cross-validation technique enhances the reliability of the results by performing training and accuracy assessment multiple times. As listed in Table 3, the ensemble model **IV** exhibited an accuracy of 78.60% and an AUC of 83.93% under 10-fold cross-validations. Additionally, other performance metrics including sensitivity, precision, F1 score, and specificity were comparable to the performance exhibited by this model without cross-validation (Table 2). The primary criterion to evaluate the performance of any model is the predictive performance metrics, as they are considered quantifiable to be used as a standard for the evaluation of the model. It demonstrated a sensitivity of 94.52%, precision of 80.14%, and f1 score of 85.62%, obtained by the 10-fold cross-validation, being comparable to those without cross-validation (Table 2). The results thereby justified the reliability of our models.

**TABLE 3:** Performance metrics of base classifiers and ensemble model **IV** built on Morgan fingerprints using 10-fold cross-validation.

Models	Accuracy	AUC	F1 score	Sensitivity	Specificity	Precision	BA
**KNN**	0.7151 ± 0.0294	0.6733 ± 0.0479	0.8196 ± 0.0169	0.9856 ± 0.0192	0.1657 ± 0.0113	0.7019 ± 0.0229	0.575 ± 0.0405
**SVC**	0.7589 ± 0.0493	0.8010 ± 0.08164	0.8333 ± 0.03603	0.9078 ± 0.0513	0.4628 ± 0.0142	0.7709 ± 0.0328	0.6847 ± 0.0550
**RF**	0.7417 ± 0.0468	0.7661 ± 0.0948	0.8194 ± 0.0328	0.9221 ± 0.0449	0.421 ± 0.0184	0.7548 ± 0.0359	0.6512 ± 0.0531
**ET**	0.7437 ± 0.0532	0.7572 ± 0.0954	0.8266 ± 0.0315	0.9051 ± 0.0363	0.4457 ± 0.0218	0.7689 ± 0.0409	0.6842 ± 0.0686
**RNN**	0.7432 ± 0.0112	0.7939 ± 0.0095	0.8024± 0.0086	0.7836 ± 0.0098	0.6628 ± 0.02044	0.8221 ± 0.0097	0.7232 ± 0.0130
**MLP**	0.6968 ± 0.0182	0.7268 ± 0.0186	0.7690± 0.0172	0.8237 ± 0.0392	0.5302 ± 0.0415	0.7222 ± 0.0142	0.6919 ± 0.0129
**Ensemble model IV**	**0.7860 ±** **0.0314**	**0.8393 ±** **0.0561**	**0.8562±** **0.0365**	**0.9452 ±** **0.02904**	0.4529 ± 0.1349	0.8014 ± 0.0267	0.7038 ± 0.0754

Note: The ensemble model **IV**, is built on 4 ML, models (KNN, SVC, RF, ET) and RNN, base models. This performance of ensemble model **IV**, is based on 10-fold cross-validation. MLP, base classifier is based on the Morgan fingerprints but is not included in the above ensemble model **IV**. Best scores are marked as bold.

After achieving the best performance of the ensemble model, consisting of 4 ML models combined with RNN, we tested additional DL models to assess the ensemble performance. This time we opted for a multilayer perceptron (MLP) model rather than an RNN as it can more efficiently discern complex patterns and correlations within datasets because it contains several layers of interconnected nodes (Ahsan et al., 2020). The idea was to assess its performance alone or in combination with ML models using voting ensemble learning and to compare it with the above ensemble model **IV**. The ensemble model **IVB** (Table 2) containing MLP along with 4 ML models displayed slightly lower performance than the ensemble model **IV** comprising RNN. In addition, we constructed another new ensemble model **IVC** using an ensemble algorithm to incorporate MLP and RNN along with the 4 ML models. However, the performance of the ensemble model **IVC** (Table 2) comprising two DL models alongside 4 ML models was commendable but failed to surpass the outcomes of the ensemble model **IV** featuring only RNN as the DL model. We deployed a new benchmark approach utilizing different molecular descriptors/fingerprints and ML models trained on our dataset approach, which was employed in previous studies. We applied our voting ensemble method after training ML base models and compared the analysis with the published researches. One such reported study utilized the averaging ensemble method by fusing 3 ML base models constructed on twelve different descriptors (Ai et al., 2018). Similarly, another study used a neural network framework to ensemble five different base models built on different molecular features (Li et al., 2020). We successfully extracted some of the descriptors for our dataset mentioned in the above studies and constructed similar ML models. The same voting ensemble technique was employed as we used for our model, demonstrating much better performance than the published studies (Supplementary Tables S8, S9). Based on the above findings, it is evident that our proposed model has great potential in addressing and identifying the challenge of liver injury caused by chemicals and drugs. Moreover, it can assist in decreasing drug attrition rates and minimizing the risk of exposure to agents causing liver toxicity.

## Discussion

Drug-induced liver injury poses a substantial risk in the development of drugs. Many drugs failed during clinical trials while some are withdrawn from the market due to concerns of liver injury. Similarly, industrial chemicals and solvents present a severe hazard for the development of liver injury. Extensive studies have been done to predict the risk of drugs causing hepatotoxicity but the performance of these predictions falls short of optimal standards, necessitating further work to address this problem. In the current study, we proposed an ensemble model integrating ML and DL models to enhance the predicting power of the model to forecast hepatotoxicity. Ensemble model combining ML and DL is an innovative approach that influences individual models to enhance their performance and capabilities, helping in solving complex tasks across diverse domains. An extensive search was done to collect compounds from different databases and literature to get a larger dataset. To the best of our knowledge, no previous study has assembled such a large dataset of hepatotoxic compounds. Increasing sample size can offer certain benefits in enhancing the prediction accuracy of the models and mitigating the under-fitting issue stemming from the limited size of the dataset (Zanette et al., 2018). Four ensemble models were developed based on two different descriptors (including molecular descriptors, and Mordred descriptors) and Morgan fingerprints. The primary approach of a QSAR model relies heavily on the utilization of descriptors to characterize the molecular structure of biologically active compounds. The importance of different features can be assessed and quantified by leveraging information acquired from models. In current studies, some of Mordred descriptors were not generated leading to missing values, which were later deleted to avoid any kind of irregularities in the model development. A connection between the important descriptors and the model performance can be assessed from the model information and it further allows us to integrate the relationship between predictors (Kelleci Celik and Karaduman, 2023).

Initially, individual ML and DL base models were developed for each descriptor and fingerprint. The performance of the base models was unsatisfactory and improved by the feature selection approach based on the model contribution. Selecting important features from the training set could give accurate, informative, and robust performance of the model (Pradeep et al., 2021; Kelleci Celik and Karaduman, 2023). Moreover, choosing descriptors based on their model performance needs to endorse and guarantee that the selected descriptors can generalize and perform well to unseen data (Cherkasov et al., 2014). The sequential feature selection approach was utilized not only to find the optimal features but also to enhance the performance and explainability of the models while minimizing the computational complexity and overfitting (Ying, 2019). The feature selection process enhanced the performance of base models and demonstrated superior performance in comparison to the reported base models (Kang and Kang, 2021) developed with a deep neural network (DNN) along with RF, SVC, and Naive Bayesian on a dataset containing 1119 compounds to predict drug-induced liver injury, in which ECFP4 (extended connectivity fingerprints 4), ECFP6, FCFP4 (functional class fingerprint 4), and FCFP6, respectively, were utilized to get the best accuracy of 0.75 for ECFP4 using 10-fold cross-validation. Our model overcame another published work, which employed 12 different molecular fingerprints for developing three kinds of ML base models of RF, SVC, and XGBoost for each fingerprint to attain an accuracy of less than 0.71 (Ai et al., 2018). The enhancement in the model performance was not solely due to the feature importance but finding the optimal parameters for each model also played a pivotal role. The feature selection process assisted us in identifying the optimal features for the base model development, aiming to get the most accurate predictive model by excluding unproductive descriptors from the selection process. As a result, the base models and ensemble model’s predictive accuracy and other performance parameters were enhanced several times. Hong et al. (2017) extracted features for the decision forest models by calculating and ranking the frequency of each Mold2 descriptor according to their frequency values to identify the most informative descriptors but still achieved lower accuracy. The lower performance of their model could be due to using a small dataset and ML models, which lack the robustness of the ensemble strategy. Yang et al. (2024) utilized six different molecular fingerprints for ML and DNN algorithms, and achieved better performance for the DNN model while using the ECFP_6 fingerprint with an accuracy of 0.713 outperforming all the ML models. DNN got superior performance over the ML models due to their robustness however, the overall performance of all models might be limited for selecting only the top ten features using the Shapley additive explanations (SHAP) algorithm. Selecting a small number of features might have overlooked the valuable information from other relevant features, resulting in a less comprehensible model.

After achieving optimal performance of the individual models, we proceeded with the voting ensemble algorithm by keeping parameters consistent with each model’s peak performance. Some heterogeneous ensemble models are reported to be built by combining ML models with the traditional averaging method. Obviously, the ensemble approach may offer some benefits to capitalize on lower computational requirements with higher diversity, thereby potentially improving overall performance (Kelleci Celik and Karaduman, 2023). Ensemble models are considered foremost to be influential, possessing properties to reduce the overfitting of the baseline models. All four models demonstrated moderate performances whereas the voting ensemble model **IV** utilizing Morgan fingerprints exhibited excellent performance, achieving an accuracy of 80.26%, AUC of 82.84%, and a sensitivity of 93.02%. In the current study, ensemble models performed much better than reported ensemble models with prediction accuracies below 0.70 by combining five base models ML algorithms of LR (logistic regression), KNN, SVC, RF, and XGBoost (extreme gradient boosting) based on three distinct descriptors (Li et al., 2020). The potential lower accuracies might be due to using the neural network approach on a small dataset of 1002 compounds, as deep learning algorithms typically require a bigger sample size to fully leverage their capabilities. Normally, ensemble learning involves training multiple baseline models to make them combine, constructing a more powerful and impressive single model in comparison to the individual baseline models (Kumar et al., 2021). We implemented a similar ensemble approach to that employed by Islam and Nahiduzzaman (2022), however, our approach was different in utilizing the DL algorithms as well. The main idea behind using the voting ensemble approach was to enhance the model accuracy by reducing the errors inherent in individual algorithms. This was achieved by combining the decisions of multiple algorithms through a voting scheme, where the final prediction is determined by the most common outcome among all algorithms. It helps to reduce the weakness of any single model by aggregating diverse perspectives of all the models and mitigating the risk of bias or overfitting associated with individual models (Maclin, 2016; Gu et al., 2021).

An exhaustive validation process was followed to substantiate the performance of ensemble models using different benchmark methods. Initially, the models were validated by comparing the performance of the individual base models with ensemble models with and without the selected features. In both cases, the performance of the voting ensemble classifier was way better than the one achieved by each base model. Furthermore, model performance was validated by external validation using a test set consisting of 20% of randomly separated data. It is crucial to assess the generalizability and reliability of the model as it provides insights into the model’s real-world performance on unseen data. Additionally, two more ensemble learning techniques of bagging and stacking ensembles were employed along with the voting ensemble classifier, figuring out the voting ensemble as the best method for combining the base models. Bagging is a data-specific algorithm that works by creating small subsets of the actual dataset, having the potential to perform well on high-performance data. The bagging method is also known as bootstrap aggregating, involving bootstrapping and aggregating algorithms to solve any data problem (Kim and Baek, 2022). Similarly, the stacking method is based on the information generated from the multiple-base models to generate a new model. Base models are initially fit on the training data and a subsequent model learns their predictions and compiles them in a way to give the best prediction (Ma et al., 2018). In continued, the 10-fold cross-validation was applied to the best model (the ensemble model **IV**) for validating its performance to be comparable with the same model without the cross-validation method, indicating the reliability of the ensemble model. Our studies indicated a superior performance in comparison to the reported ensemble model constructed based on 3 ML classifiers (Ai et al., 2018), which had an accuracy of approximately 0.715 using the averaging ensemble approach validated by a 5-fold cross-validation. The lower performance might be attributed to the averaging ensemble model, which combines the predictions of multiple base models by averaging the predicted value. This method does not enhance the performance to a significant extent but we got the average result close to the individual predictions. On the other hand, in a voting ensemble, the predictions of multiple base models are combined through a voting process and the class with the majority of votes is selected as the final prediction. Moreover, our model outperformed other studies in which ML or DL approaches were utilized to build predictive models for hepatotoxicity (Xu et al., 2015; Hong et al., 2017; Thakkar et al., 2020).

The performance of ensemble model **IV** was further affirmed by substituting RNN with MLP and the results indicated that the ensemble model **IV** employing RNN had a better performance. Next, an ensemble model using MLP and RNN as DL models was employed along with 4 ML models but it did not exhibit better results than our best model. In this context, our objective was to assess the cumulative impact of two DL models over the ensemble algorithm, since DL algorithms have the advantage of augmented data generation to enhance computational capabilities, solving complicated issues related to unstructured and varied data (Taye, 2023). Although ensemble models **IVB** and **IVC** did not perform better than ensemble model **IV**, it is worth mentioning that all ensemble approaches outperformed the individual MLP and RNN models, confirming that ensemble methodology leads to better performance than a single DL model (Ha et al., 2005). Ensemble learning entails the integration of multiple models and this collective approach leverages the diverse perspectives and strengths of each base model to achieve robustness and enhanced predictive capability (Ganaie et al., 2022). Finally, benchmark modeling was conducted against previously reported studies, revealing our ensemble approach outperformed the previous models. The voting method combines the output of individual base models in an ensemble algorithm by counting them to predict the final results based on the labels with majority votes. It offers an advantage by exhibiting reduced bias towards the base models as its effect is mitigated by the count of majority votes, ensuring a more balanced and robust prediction. Moreover, if the majority of the base models favor a particular event, it may result in the dominance of that event in the ensemble model. Thus, it was hypothesized that the performance of the ensemble model based on the majority voting of base learners would surpass the performance of the deep learning ensemble models (Ju et al., 2018).

Despite all the advantages our model offers, there are some shortcomings associated with the studies. For instance, ensemble training can improve the predictive accuracy and robustness of the model, but it may also increase the computational complexity and resource demands. Training and implementation of an ensemble model containing both ML and DL can be time-consuming, which could be impractical in all settings. Despite performing extensive benchmark modeling and validating the model by 20% of test data, it would be preferable to validate our model with an external data set. However, it was not feasible due to the limited availability of data, especially in an instance of DL to require large data for efficient training of the model. Data type and quality are critical considerations while dealing with ML models. Similar base models may not perform well on datasets of different diseases, as the performance of any model depends significantly on the type and characteristics of data used for training. In addition, a different DL model should be selected as the base model for evaluating this ensemble strategy against other diseases depending on the type of data. The performance of the ensemble model critically depends on the effectiveness of its base models, emphasizing the need for their optimization before training the ensemble model. Data imbalance can lead to a biased model, potentially affecting the learning process and reducing the model’s ability to accurately predict the minority class, impacting the overall performance of the model (Kulkarni et al., 2020). Although, our dataset was slightly imbalanced but still we got efficient predictive performance. Addressing the issue of this imbalance could potentially lead to an even better model with enhanced performance.

## Conclusion

Improving the predictive performance of models built on chemical and drug-induced hepatotoxicity is essential for the safety and efficacy of new pharmaceuticals and for accelerating the drug approval process. Each model gathers distinct data points, leading to different correlations with the endpoints and produces varying results. Our proposed ensemble model utilizes varying information generated from different data end points of individual models and makes stable predictions with higher performance than any single individual model. Robust *in silico* models hold the potential to replace animal tests, serving as an initial step in recognizing potential adverse effects of drugs on the target organ.

## Data Availability

Publicly available datasets were analyzed in this study. This data can be found here: All the datasets used are mentioned in the manuscript (links are provided where necessary).
