# Supplementary material for: Comprehensive hepatotoxicity prediction: ensemble model integrating machine learning and deep learning
Source: Front Pharmacol. 2024 Aug 21;15:1441587. doi: 10.3389/fphar.2024.1441587 (PMC11373136; doi:10.3389/fphar.2024.1441587)
Supplement: Supplementary file 1 [file Table1.DOCX]

Supplementary Material

**Table S1:** Results of the base models built on RDKit molecular descriptors.

| **Models** | **Accuracy** | **AUC** | **F1 score** | **sensitivity** | **Specificity** | **Precision** | **BA** |
| --- | --- | --- | --- | --- | --- | --- | --- |
| **KNN** | 0.6807 | 0.7163 | 0.7580 | 0.7669 | 0.5193 | 0.7492 | 0.6431 |
| **SVM** | 0.7153 | 0.7472 | 0.8026 | 0.8879 | 0.3922 | 0.7323 | 0.6400 |
| **RF** | 0.7365 | 0.7811 | 0.8083 | 0.8525 | 0.5193 | 0.7686 | 0.6859 |
| **ET** | 0.7134 | 0.7859 | 0.7910 | 0.83185 | 0.4917 | 0.7540 | 0.6617 |
| **RNN** | 0.7019 | 0.6812 | 0.7662 | 0.74926 | 0.5801 | 0.7839 | 0.6735 |
| **Ensemble model I** | 0.7286 | 0.7785 | 0.8077 | 0.8513 | 0.4806 | 0.7504 | 0.6636 |

**Table S2:** Results of the base models constructed on Mordered descriptors.

| **Models** | **Accuracy** | **AUC** | **F1 score** | **sensitivity** | **Specificity** | **Precision** | **BA** |
| --- | --- | --- | --- | --- | --- | --- | --- |
| **KNN** | 0.7151 | 0.7401 | 0.7792 | 0.7557 | 0.6342 | 0.8042 | 0.6950 |
| **SVM** | 0.7227 | 0.7585 | 0.8133 | 0.9080 | 0.3542 | 0.7365 | 0.6311 |
| **RF** | 0.7415 | 0.7934 | 0.8183 | 0.8477 | 0.5542 | 0.7908 | 0.7009 |
| **ET** | 0.7436 | 0.7939 | 0.8171 | 0.8477 | 0.5485 | 0.7887 | 0.6981 |
| **RNN** | 0.7246 | 0.7583 | 0.7942 | 0.7988 | 0.5771 | 0.7897 | 0.6879 |
| **Ensemble model II** | 0.7463 | 0.7971 | 0.8105 | 0.8377 | 0.5714 | 0.7851 | 0.7167 |

**Table S3:** Results of the base models using RDKit molecular descriptors and Mordered descriptors combine

| **Models** | **Accuracy** | **AUC** | **F1 score** | **sensitivity** | **Specificity** | **Precision** | **BA** |
| --- | --- | --- | --- | --- | --- | --- | --- |
| **KNN** | 0.6519 | 0.6261 | 0.7454 | 0.7817 | 0.4088 | 0.7123 | 0.5952 |
| **SVM** | 0.7192 | 0.7170 | 0.8147 | 0.9469 | 0.2928 | 0.7149 | 0.6198 |
| **RF** | 0.7153 | 0.7120 | 0.8010 | 0.8790 | 0.4088 | 0.7358 | 0.6439 |
| **ET** | 0.7019 | 0.7141 | 0.7919 | 0.8702 | 0.3867 | 0.7266 | 0.6284 |
| **RNN** | 0.6653 | 0.6223 | 0.7485 | 0.7640 | 0.4806 | 0.7337 | 0.6223 |
| **Ensemble model III** | 0.7192 | 0.7167 | 0.8058 | 0.8938 | 0.5308 | 0.7336 | 0.6523 |

**Table S4:** Results of the base models constructed on Morgan's fingerprints.

| **Models** | **Accuracy** | **AUC** | **F1 score** | **sensitivity** | **Specificity** | **Precision** | **BA** |
| --- | --- | --- | --- | --- | --- | --- | --- |
| **KNN** | 0.724 | 0.702 | 0.808 | 0.905 | 0.3371 | 0.736 | 0.621 |
| **SVM** | 0.731 | 0.768 | 0.815 | 0.902 | 0.44 | 0.744 | 0.654 |
| **RF** | 0.737 | 0.7928 | 0.827 | 0.873 | 0.525 | 0.785 | 0.699 |
| **ET** | 0.735 | 0.779 | 0.822 | 0.853 | 0.561 | 0.794 | 0.706 |
| **RNN** | 0.682 | 0.722 | 0.7529 | 0.727 | 0.532 | 0.780 | 0.66 |
| **Ensemble model IV** | 0.7686 | 0.7949 | 0.8438 | 0.9086 | 0.541 | 0.7658 | 0.6941 |

**Table S5:** Results of the base models constructed on RDKit molecular descriptors using the feature extraction process.

| **Models** | **Accuracy** | **AUC** | **F1 score** | **sensitivity** | **Specificity** | **Precision** | **BA** |
| --- | --- | --- | --- | --- | --- | --- | --- |
| **KNN** | 0.7076 | 0.7337 | 0.7809 | 0.7994 | 0.5359 | 0.7633 | 0.6676 |
| **SVM** | 0.7269 | 0.7640 | 0.8111 | 0.8997 | 0.4033 | 0.7384 | 0.6515 |
| **RF** | 0.7461 | 0.7971 | 0.8191 | 0.8820 | 0.4917 | 0.7647 | 0.6868 |
| **ET** | 0.7192 | 0.7890 | 0.7937 | 0.8289 | 0.5138 | 0.7615 | 0.6713 |
| **RNN** | 0.7365 | 0.7818 | 0.8045 | 0.8318 | 0.5580 | 0.7790 | 0.6949 |
| **Ensemble model I** | 0.7381 | 0.7893 | 0.8107 | 0.8521 | 0.5189 | 0.7657 | 0.6871 |

**Table S6:** Results of the base models constructed on Mordered descriptors using the feature extraction process.

| **Models** | **Accuracy** | **AUC** | **F1 score** | **sensitivity** | **Specificity** | **Precision** | **BA** |
| --- | --- | --- | --- | --- | --- | --- | --- |
| **KNN** | 0.7189 | 0.7432 | 0.7866 | 0.7787 | 0.6 | 0.7947 | 0.6893 |
| **SVM** | 0.7361 | 0.7630 | 0.8226 | 0.9195 | 0.3714 | 0.7441 | 0.6454 |
| **RF** | 0.7629 | 0.8015 | 0.8277 | 0.8563 | 0.5771 | 0.8010 | 0.7167 |
| **ET** | 0.7743 | 0.8181 | 0.8396 | 0.8879 | 0.5485 | 0.7963 | 0.7182 |
| **RNN** | 0.7342 | 0.7574 | 0.8005 | 0.8017 | 0.6 | 0.7994 | 0.7008 |
| **Ensemble model II** | 0.7589 | 0.7910 | 0.8227 | 0.8613 | 0.5448 | 0.7867 | 0.7081 |

**Table S7:** Results of the base models constructed combining RDKit molecular descriptors and Mordered descriptors using the feature selection process

| **Models** | **Accuracy** | **AUC** | **F1 score** | **sensitivity** | **Specificity** | **Precision** | **BA** |
| --- | --- | --- | --- | --- | --- | --- | --- |
| **KNN** | 0.7151 | 0.7359 | 0.7868 | 0.7902 | 0.5657 | 0.7834 | 0.6779 |
| **SVM** | 0.7189 | 0.7528 | 0.8112 | 0.9080 | 0.3428 | 0.7331 | 0.6254 |
| **RF** | 0.7571 | 0.8009 | 0.8243 | 0.8563 | 0.5600 | 0.7946 | 0.7081 |
| **ET** | 0.7456 | 0.7932 | 0.8190 | 0.8649 | 0.5085 | 0.7777 | 0.6867 |
| **RNN** | 0.7189 | 0.7429 | 0.7860 | 0.7758 | 0.6057 | 0.7879 | 0.6907 |
| **Ensemble model III** | 0.7532 | 0.7921 | 0.8242 | 0.8713 | 0.5224 | 0.7808 | 0.6967 |

**Tabel S8.** Benchmark training comparison of our model with the previously published studies using the MACCs descriptors.

| **Models** | **LR** | | **KNN** | | **SVM** | | **RF** | | **XGBoost** | | **Ensemble Results** | |
| --- | --- | --- | --- | --- | --- | --- | --- | --- | --- | --- | --- | --- |
|  | **Deep** | **Ense** | **Deep** | **Ense** | **Deep** | **Ense** | **Deep** | **Ense** | **Deep** | **Ense** | **Deep** | **Ense** |
| **MCC** | 0.198 | 0.259 | 0.110 | 0.344 | 0.098 | 0.324 | 0.177 | 0.324 | 0.120 | 0.312 | **0.267** | **0.368** |
| **accuracy** | 0.606 | 0.668 | 0.589 | 0.701 | 0.601 | 0.700 | 0.611 | 0.697 | 0.589 | 0.684 | **0.647** | **0.715** |
| **AUC** | 0.636 | 0.690 | 0.586 | 0.665 | 0.611 | 0.729 | 0.643 | 0.649 | 0.616 | 0.723 | **0.640** | **0.768** |
| **F1 score** | 0.657 | 0.754 | 0.685 | 0.771 | 0.721 | 0.784 | 0.686 | 0.777 | 0.678 | 0.755 | **0.703** | **0.789** |
| **BA** | 0.601 | 0.620 | 0.551 | 0.665 | 0.538 | 0.642 | 0.587 | 0.648 | 0.557 | 0.652 | 0.634 | 0.662 |
| **sensitivity** | 0.630 | 0.811 | 0.747 | 0.805 | 0.859 | 0.871 | 0.710 | 0.842 | 0.722 | 0.778 | 0.698 | 0.881 |
| **specificity** | 0.571 | 0.429 | 0.355 | 0.526 | 0.217 | 0.412 | 0.463 | 0.455 | 0.393 | 0.526 | 0.570 | 0.442 |

**Deep:** Deep in the table represent the model **previously performed** while **Ense** represents the models trained on our dataset with the MACCs descriptors. All the results of our models (Ense models) both individual and ensemble models are based on 5-fold cross validation to compare it with the published studies.

**Tabel S9.** Benchmark training comparison of our model with the previously published studies using the Mold2 descriptors

| **Models** | **LR** | | **KNN** | | **SVM** | | **RF** | | **XGBoost** | | **Ensemble Results** | |
| --- | --- | --- | --- | --- | --- | --- | --- | --- | --- | --- | --- | --- |
|  | **Deep** | **Ense** | **Deep** | **Ense** | **Deep** | **Ense** | **Deep** | **Ense** | **Deep** | **Ense** | **Deep** | **Ense** |
| **MCC** | 0.130 | 0.284 | 0.125 | 0.310 | 0.220 | 0.316 | 0.225 | 0.343 | 0.219 | 0.333 | **0.331** | **0.405** |
| **accuracy** | 0.617 | 0.671 | 0.582 | 0.684 | 0.646 | 0.698 | 0.645 | 0.707 | 0.642 | 0.699 | **0.687** | **0.728** |
| **AUC** | 0.628 | 0.700 | 0.580 | 0.651 | 0.645 | 0.716 | 0.658 | 0.651 | 0.651 | 0.727 | **0.659** | **0.779** |
| **F1 score** | 0.744 | 0.745 | 0.657 | 0.756 | 0.752 | 0.787 | 0.736 | 0.789 | 0.732 | 0.774 | **0.755** | **0.801** |
| **BA** | 0.54 | 0.639 | 0.562 | 0.651 | 0.584 | 0.633 | 0.600 | 0.651 | 0.598 | 0.657 | 0.658 | 0.680 |
| **sensitivity** | 0.932 | 0.766 | 0.669 | 0.782 | 0.899 | 0.890 | 0.828 | 0.873 | 0.818 | 0.823 | 0.805 | 0.888 |
| **specificity** | 0.147 | 0.511 | 0.454 | 0.519 | 0.268 | 0.375 | 0.371 | 0.429 | 0.378 | 0.491 | 0.510 | 0.472 |

**Deep:** Deep in the table represent the model previously performed while **Ense** represents the models trained on our dataset with the Mold2 descriptors. All the results of our models (Ense models) both individual and ensemble models are based on 5-fold cross validation to compare it with the published studies.
